# Beyond the Brain: Perinatal Exposure of Rats to Serotonin Enhancers Induces Long-Term Changes in the Jejunum and Liver

**DOI:** 10.3390/biomedicines12020357

**Published:** 2024-02-03

**Authors:** Romana Gračan, Sofia Ana Blažević, Matea Brižić, Dubravka Hranilovic

**Affiliations:** 1 Division of Zoology, Department of Biology, Faculty of Science, University of Zagreb, 10000 Zagreb, Croatia; romana.gracan@biol.pmf.unizg.hr; 2 Division of Animal Physiology, Department of Biology, Faculty of Science, University of Zagreb, 10000 Zagreb, Croatia; matea305@gmail.com (M.B.); dubravka.hranilovic@biol.pmf.unizg.hr (D.H.)

**Keywords:** serotonin, 5-hydroxytryptophan, tranylcypromine, monoamine oxidase, liver, jejunum, histological techniques, qRT-PCR, enterochromaffin cells, rat

## Abstract

Serotonin (5-hydroxytryptamine, 5HT) homeostasis is essential for many physiological processes in the central nervous system and peripheral tissues. Hyperserotonemia, a measurable sign of 5HT homeostasis disruption, can be caused by 5HT-directed treatment of psychiatric and gastrointestinal diseases. Its impact on the long-term balance and function of 5HT in the peripheral compartment remains unresolved and requires further research due to possible effects on human health. We explored the effects of perinatal 5HT imbalance on the peripheral organs responsible for serotonin metabolism—the jejunum, a synthesis site, and the liver, a catabolism site—in adult rats. Hyperserotonemia was induced by subchronic treatment with serotonin precursor 5-hydroxytryptophan (5HTP) or serotonin degradation inhibitor tranylcypromine (TCP). The jejunum and liver were collected on postnatal day 70 and analyzed histomorphometrically. Relative mRNA levels of 5HT-regulating proteins were determined using qRT-PCR. Compared to controls, 5HTP- and TCP-treated rats had a reduced number of 5HT-producing cells and expression of the 5HT-synthesising enzyme in the jejunum, and an increased expression of 5HT-transporter accompanied by karyomegaly in hepatocytes, with these differences being more pronounced in the TCP-treated animals. Here, we report that perinatal 5HT disbalance induced long-term cellular and molecular changes in organs regulating 5HT-metabolism, which may have a negative impact on 5HT availability and function in the periphery. Our rat model demonstrates a link between the developmental abnormalities of serotonin homeostasis and 5HT-related changes in adult life and may be suitable for exploring the neurobiological substrates of vulnerability to behavioral and metabolic disorders, as well as for modeling the adverse effects of the prenatal exposure to 5HT enhancers in the human population.

## 1. Introduction

Serotonin (5-hydroxytryptamine, 5HT) is a biologically active amine present in the central nervous system (CNS) and the peripheral tissues of mammals. In the “central 5HT compartment”, it is best known as a neurotransmitter that modulates neural activity and a range of neuropsychological processes including mood, perception, reward, anger, aggression, appetite, memory, cognition, pain sensitivity, thermoregulation, sleep, sexual behavior, and circadian rhythm [1,2]. The cell bodies of 5HT-synthesizing neurons are located in the raphe nuclei of the brain stem and project their axons throughout the cortical and subcortical regions of the brain. The first step in serotonin biosynthesis, the conversion of L-tryptophan to 5-hydroxytryptophan (5HTP), is catalyzed by the enzyme tryptophan hydroxylase (TPH2 isoform), which is followed by the reduction to 5HT via aromatic L-amino acid decarboxylase and active transport into synaptic vesicles by vesicular monoamine transporter (VMAT2 isoform). 5HT synaptic action is terminated through its reuptake by 5HT transporter (5HTt) and degradation to 5-hydroxyindoleacetic acid by the enzyme monoamine oxidase (MAO), preferentially the MAOA isoform [3].

Nonetheless, most serotonin is found outside the CNS, in the “peripheral compartment”. 5HT is primarily produced in the gut enterochromaffin (EC) cells of the mucosa [4] by the TPH1 isoform and packed into dense granules by the VMAT1 isoform [5]. Upon its release into circulation, platelets accumulate the majority of peripheral serotonin, while small amounts of 5HT remaining in platelet-free plasma can activate more than seventeen 5HT receptors located in various peripheral tissues [6] before 5HT is taken up by 5HTt and catabolized by MAOA in the endothelial cells of the lungs and liver [7]. Peripheral 5HT regulates numerous biological processes in cardiovascular, pulmonary, gastrointestinal (GI), and genitourinary systems [1]. 5HT from the circulatory system governs hemodynamics, modifies blood pressure, and changes body temperature through cutaneous vasodilation [4,7]. Previous studies have suggested that EC cells are pressure sensors that secrete serotonin into the wall of the GI tract, initiate peristaltic and secretory reflexes, and activate sensory nerves [8]. However, recent studies imply that 5HT from the neurons of the enteric system is more important for constitutive gastrointestinal transit than is enteric serotonin from EC cells. Neuronal 5HT also promotes the growth/maintenance of the mucosa and neurogenesis, while enteric serotonin induces a GI inflammatory response [9]. Enteric serotonin enters the liver through the portal vein and affects several key processes: gluconeogenesis [6], hepatic lipid metabolism via a gut–liver endocrine axis, hepatic blood flow (portal and sinusoidal), regeneration, innervation, and wound healing [10,11].

The central and the peripheral serotonergic systems are functionally separated by a blood–brain barrier and regulated independently. However, during the fetal and early postnatal development of the brain, the blood–brain barrier is not fully formed, and the two systems can freely communicate [12]. Considering the role of 5-HT in neurodevelopment [13] and prenatal programming [14], genetic or environmental disruption of optimal serotonin concentrations can affect serotonin homeostasis in both compartments, leading to an increased vulnerability to behavioral and/or metabolic disorders [15,16]. One of the measurable signs of 5HT homeostasis disruption is hyperserotonemia—a state of elevated blood 5HT levels. It has been identified in some developmental disorders such as those of autism spectrum but can also be caused by the use of serotonergic medications developed for medical treatments of psychiatric, neurological, and some gastrointestinal disorders [17,18,19]. In addition to the “classical” 5HT-targeting drugs, such as MAO inhibitors, selective serotonin reuptake inhibitors (SSRIs), or agents targeting specific serotonin receptors, the immediate 5HT precursor 5-HTP has been widely offered for alleviating depressive symptoms, binge eating, headache, or insomnia [20]. Although the effects of serotonergic drugs have been examined in detail and are fully understood in the CNS and partially in the digestive tract [21], some side effects (e.g., diabetes, metabolic syndrome, and valvular heart disease [8,22]) reveal that the physiological role of the peripheral serotonergic system is still unclear and needs to be studied further. This especially holds true for 5HTP, whose safety of use has hardly been studied in the human population or in animal models [23].

To identify the consequences of developmentally disturbed serotonin homeostasis, we pharmacologically induced elevated blood 5HT levels by the perinatal treatment of rats with serotonin precursor 5-hydroxytryptophan (5HTP) or MAO inhibitor tranylcypromine (TCP) during the most intensive development of 5HT neurons. The aim was to induce perinatal hyperserotonemia through endogenous serotonin, i.e., not through an analog but through the inhibition of catabolism or the stimulation of synthesis at two levels. First, primarily at the peripheral level, the administration of 5-hydorxytryptophan, the immediate precursor of 5HT, allowed us to bypass the rate-limiting step in the synthesis of 5HT and significantly increase peripheral serotonin (treatment explained in more detail in [24]). Second, both at the central and peripheral levels, TCP inhibited both monoamine oxidase isoforms inducing long-term hyperserotonemia (explained in more detail in [25]). In our previous studies, we showed the immediate effects of the treatments and their long-lasting impact on the central 5HT compartment at the molecular, neurochemical, and behavioral level. While treatment with 5HTP significantly raised peripheral but not central 5HT concentrations, treatment with TCP induced significant 5HT elevations in both “compartments” [26]. Still, both treatments increased pup mortality, reduced weight gain, compromised thermoregulation, and altered affiliative behavior [24,25,27]. At adult age, 5HTP-treated rats displayed a modest but significant decrease in 5HT concentration [26] and an increase in *MaoA* and *MaoB* mRNA abundance [28] in the frontal cortex, accompanied by increased exploratory activity [29]. More prominent long-lasting effects were observed in TCP-treated rats. Disturbed peripheral 5HT homeostasis was reflected in hyperserotonemia, altered bone remodeling, and hematopoiesis [30]. Disturbed central 5HT homeostasis was reflected in a significant increase in mRNA abundance for both *Mao* genes [28] and, consequently, increased brain 5HT metabolism and drastically decreased 5HT concentrations in the frontal cortex and raphe nuclei [26], accompanied by highly anxiolytic behavior [29].

In this study, we further explored the structural and molecular backgrounds of the disturbed peripheral homeostasis by focusing on the peripheral organs responsible for serotonin metabolism—the digestive tract, where enteric 5HT is synthesized, and the liver, where enteric 5HT is primarily metabolized. Our aim was to analyze the long-lasting histomorphological and 5HT-regulating gene expression changes in the jejunum and liver tissue after 5HTP-induced and TCP-induced perinatal hyperserotonemia. Jejunum and liver samples were collected from 13 5HTP-treated, 13 TCP-treated, and 11 saline-treated rats of both sexes on postnatal day (P) 70; the samples were analyzed histomorphometrically; and the relative expression of the genes coding for TPH1 and VMAT1 in the jejunum and 5HTt and MAOA in the liver was compared among the three groups.

## 2. Materials and Methods

We performed a two-step chronic treatment of animals with either of the two 5HT-enhancers—5-hydorxytryptophan (5HTP) and tranylcypromine (TCP)—or with saline. In the first step, we treated pregnant females (3 with each of the 5HT enhancers and 2 with saline) from gestational day 12 until parturition. In the second step, we treated pups (13 with TCP, 13 with 5HTP, and 11 with saline) from postnatal day (PND) 1 until PND21. At PND70, blood, jejunum, and liver samples were collected from all experimental and control rats. Serum 5HT concentrations were measured with ELISA, jejunum and liver tissue was examined histomorphologically, and the relative expression of the genes of the serotonin pathway proteins, including tryptophan hydroxylase 1 (*Tph1*) and vesicular monoamine transporter 1 (*Vmat1*)—responsible for serotonin synthesis and storage in the jejunum—and serotonin transporter (*5HTt*) and monoamine oxidase A (*MaoA*)—responsible for 5HT elimination in liver—were determined via quantitative PCR.

### 2.1. Housing and Breeding of Animals

The experiment was performed on eight nulliparous Wistar females acquired from the animal facility of the Croatian Institute for Brain Research (University of Zagreb, Zagreb, Croatia), weighing 220–250 g, which were randomly assigned to a saline, 5-hydroxytryptophan (5HTP), or tranylcypromine (TCP) group and mated with males of the same strain and age in a 3:1 or 2:1 ratio in order to synchronize paring and parturition [31] and reduce the number of male progenitors in accordance with the 3R’s principle [32]. Nulliparous females were used in order to eliminate the effect of previous pregnancies and lactation on the dam’s affiliative behavior influenced by serotonin [33] through the HPA axis [34] and to allow for the same conditions for all pups being reared. After gravidity was confirmed in all females, the male was removed from the cage. Females remained together until 2 days before parturition when they were separated and remained singly housed until weaning of the pups. After weaning, animals were kept 3–4 per cage in polycarbonate cages under 12 h light:12 h dark conditions at a temperature of 22 ± 2 °C, with free access to rat chow and tap water. Animals were kept at the Animal facility of the Division of Animal Physiology, Department of Biology, Faculty of Science, University of Zagreb (Facility reg. num. HR-POK-027).

The animals’ health status was monitored throughout the experiments by a health surveillance program according to Federation of European Laboratory Animal Science Associations (FELASA) guidelines. The rats were free of all viral, bacterial, and parasitic pathogens listed in the FELASA recommendations. All efforts were made to reduce the number of animals used and to minimize animal suffering. The study was approved by the ethics committee of the University of Zagreb (251-58-508-10-19) and was conducted in accordance with the Directive of The European Parliament and of the Council (2010/63/EU) and the Croatian Animal Protection Law (NN, 102/2017, NN 32/2019) as well as the directive on animal protection in scientific research (NN 55/2013, NN, 116/2019).

### 2.2. Pharmacological Treatments

Rats were treated with serotonin synthesis precursor, 5-hydroxytryptophan (5HTP), or with the nonselective MAO inhibitor, tranylcypromine (TCP), in order to increase the level of serotonin and induce hyperserotonemia. The treatment started prenatally from the 12th until the 21st gestation day by treating pregnant females—three with 2 mg/kg of TCP (Sigma–Aldrich, St. Louis, MO, USA), three with 25 mg/kg of 5HTP (Sigma–Aldrich), and two with saline. The experiment continued with postnatal treatment of pups from P1 to P21—13 rats with TCP, 13 rats with 5HTP, and 11 rats with saline (control group). Young adult rats of both sexes were used to check for the potential differences in vulnerability to disbalance in 5HT homeostasis and avoid a sex-biased interpretation of the results [35]. Solutions were delivered in volumes of 1.51 mL per kg of body mass to dams, in volumes of 3.3 mL per kg of body mass to pups until they reached 15 g, and in volumes of 5 mL per kg of body mass until the end of treatment. The control group was treated with saline in the same manner. All subcutaneous injections were performed between 2 and 3 pm. 5HTP was dissolved in acidified saline, neutralized with NaOH, and warmed to body temperature, while TCP was dissolved in ethanol and saline, neutralized with HCl, and again warmed to body temperature.

### 2.3. Collection and Processing of Tissue Samples

On P 70 ± 1, all 37 rats were decapitated under isofluorane anesthesia (C3H2ClF5O; Mr = 184.49 g/mol; Baxter, Deerfield, IL, USA). Blood samples were collected as reported in Blazevic et al. [30] and analyzed as reported in our previous study. Tissue samples were collected for histological and gene expression analysis from the second part of the small intestine (jejunum) and from the left liver lobe. For mRNA expression analysis, approximately 85 mg of the liver and 65 mg of jejunum tissue were immediately cut with a scalpel, washed in cold saline, placed in microtubes, and frozen in liquid nitrogen. Samples for histological processing were fixed in 10% neutral formalin for 24 h, dehydrated through graded alcohol series (70–100%), cleared in xylene, and embedded in Paraplast embedding media (Sherwood Medical, Norfolk, NE, USA). Cross sections were cut on a rotating microtome (Shandon Finesse 325, Thermo Fisher Scientific, Waltham, MA, USA) at 5–7 µm in thickness, stained with hematoxylin–eosin (HE), and processed for general histology examination and analysis. Additionally, small intestine sections were stained with the histochemical Masson–Fontana method to identify neuroendocrine argentaffin cells, while liver sections were stained with periodic acid–Schiff (PAS) to highlight basement membranes and enable precise morphometry. The tissue samples from all of the groups were coded and studied independently in a blinded fashion, with 1–2 sections being selected at random for each rat.

### 2.4. qRT-PCR

Samples were disrupted and homogenized with an ultrasonic homogenizer (Bandelin electronic, Mecklenburg-Vorpommern, Germany) in 500 µL of guanidinium thiocyanate solution and frozen at −80 °C until further processing. RNA isolation was performed utilizing the phenol-free RNAqueous-4PCR kit (Ambion Inc., Austin, TX, USA), following the manufacturer’s guidelines. Subsequently, genomic DNA was eliminated as per the provided instructions. RNA quality and concentrations were assessed through agarose gel (1.5%) electrophoresis and measured in a spectrophotometer (Biochrome). Samples with degraded RNA or 260/280 nm ratios outside the 1.7–2.1 range were excluded from further processing (Supplementary Materials). Following the manufacturer’s instructions, mRNA was reversely transcribed from 1 µg of total RNA using MuLV reverse transcriptase (Applied Biosystems, Foster City, CA, USA) and oligo dT primers (Applied Biosystems, Foster City, CA, USA). The efficacy of reverse transcription was assessed through end-point PCR with the primers provided in the kit. Until further analysis, cDNA was stored at −20 °C.

Relative expression was assessed through qPCR using the TaqMan gene expression master mix (Applied Biosystems, Foster City, CA, USA) according to the manufacturer’s instructions for the following genes of interest (all primer probe sets predesigned and acquired from Applied Biosystems): tryptophan hydroxylase (*Tph1*, Rn01476869_m1), monoamine oxidase A (*MaoA*, Rn01430961_m1), vesicle-monoamine transporter (*Vmat*, Rn00564688_m1), and serotonin transporter (*5HTt*, Rn00564737_m1). Serial dilutions were employed to validate primers, duplex reactions, and ascertain the initial concentrations of cDNA. All reactions were performed in a duplex setup with primer limited rat β-actin (ACTB, VIC labelled, 4352340E, Applied Biosystems) or glyceraldehyde 3-phosphate dehydrogenase (GAPDH, VIC labelled, 4352338E, Applied Biosystems) as endogenous control reference genes (REF) and completed in duplicate. GOIs were amplified in duplex with each REF gene separately. Pipetting errors were minimized and efficiency maximized using duplexing, reducing the need for multiple technical replicates, especially with limited samples.

The qPCR setup on the AB 7300 real-time PCR System involved a two-minute incubation at 50 °C, followed by 10 min at 95 °C, and then 40 cycles of 95 °C for 15 s and 60 °C for 60 s. Amplification results were analyzed using 7300 System SDS v1.4. software (Applied Biosystems, Foster City, CA, USA). Relative gene expression was calculated according to the Pfaffl method with efficiency (E) correction [36] according to the following equation: (EGOI)∆Ct GOI/Geometric Mean [(EREF)∆Ct REF]. Experiments were conducted following the Minimum Information for Publication of Quantitative Real-Time PCR Experiments (MIQE) guidelines [37]. *Tph1* and *MaoA* gene expression was analyzed and partially reported (for saline and TCP) in our previous study [30], in which we applied a different method for calculating relative gene expression.

### 2.5. Quantitative Histomorphometric Analysis

To determine whether experimentally caused hyperserotonemia increased the number of argentaffin cells which produce serotonin in the mucosal epithelial layer, we counted the number of positive cells stained with the specific Masson–Fontana method. The whole digestive tract surface (without lumen) was measured, and cells were counted on 1 cross section of the jejunum in the surface epithelial and crypt epithelial area separately. These values were expressed as the average number of argentaffin cells on 1 mm of referent space. In some cases, there was a positive reaction in lymphocytes, erythrocytes, and Paneth cells, which were all excluded from counting.

**Figure 1:**
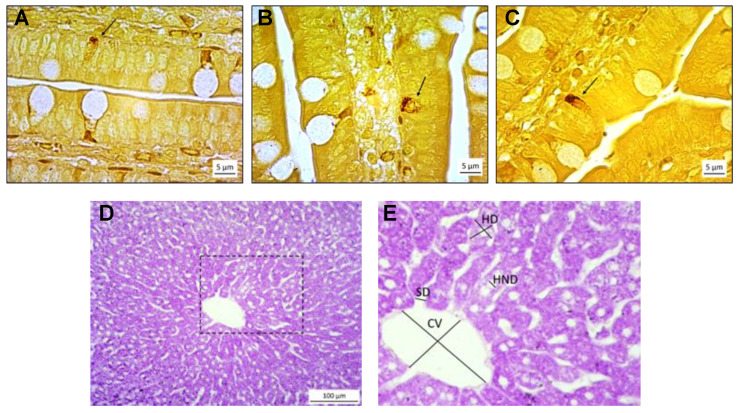
Histological sections of the jejunum with three representative argentaffin (serotonin-producing) cells (arrow) of different staining intensity: (**A**) low staining intensity, (**B**) medium staining intensity, and (**C**) high staining intensity (Masson–Fontana technique, magnification: 1000×). Normal histological structure of the (**D**) hepatic lobule in the rat liver with a rectangular area enlarged at (**E**) showing analyzed histomorphometric parameters. CV—central vein; HND—hepatocyte nuclei diameter; HD—hepatocyte diameter; SD—sinusoid diameter. PAS stain, magnification: 100×.

To determine the quantity of argentaffin granules in positive cells, we examined each cell with 1000× magnification, assigned the values from 1 (low intensity of stain) to 3 (high intensity of stain), and averaged these results for each animal (Figure 1A–C). We also measured the thickness of the tunica mucosa, the height of the mucosal epithelial cells, and the thickness of the outer muscle wall (tunica muscularis) at 5 randomly chosen test fields at magnification of 100× for each animal.

To investigate whether altered serotonin homeostasis affected structures in the liver, we morphometrically analyzed 10 randomly chosen test fields (at a magnification of 200×) per animal and digitally captured the pericentral zone around the central vein in hepatic lobules from 34 liver samples stained with PAS. In each field, we measured the following: the minimum and maximum width of 10 hepatocytes, the width of 10 hepatocyte nuclei, the diameter of 10 sinusoids, and the minimum and maximum diameter of a central vein (Figure 1D,E). All morphometric measures were performed with a computerized image analysis system comprised of a light microscope (Nikon Eclipse E600), a digital camera (AxioCamErc 5s, Zeiss), and ZENlite 2.1 software (Carl Zeiss Microscopy, GmbH, Jena, Germany).

### 2.6. Statistical Analysis

Data were processed with GraphPad Prism 9.1.2 software (GraphPad Software, Inc., La Jolla, CA, USA). The normality of distribution was checked with Kolmogorov–Smirnov test. Original or transformed values of all parameters were analyzed with two-way ANOVA with treatment and sex as the main effects. Tukey’s multiple comparison test was used for post hoc analyses. Correlation was analyzed with Pearson r correlation factor on all measured parameters. Values are presented as the median with interquartile range. The level of significance was set to 0.05 (two-tail *p* value). Some samples were lost during processing, and a female TCP-treated rat was excluded from all analyses, as the results were consistently outliers. The final number of samples on which the statistical analyses were performed is given in Table 1.

## 3. Results

### 3.1. Blood 5HT Concentrations

**Figure 2:**
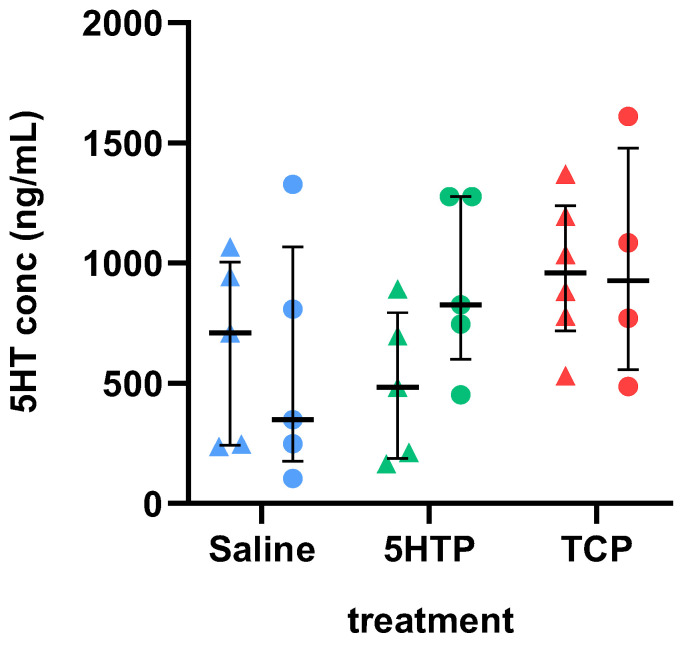
Effects of chronic treatment with 25 mg/kg 5-hydroxytryptophan (5HTP) or 2 mg/kg tranylcypromine (TCP) on 5HT concentrations in whole blood expressed as ng 5HT per mL of blood. Results are shown as the median with interquartile range; males are depicted as triangles and females as circles.

Although blood 5HT concentrations were analyzed and partially reported (for saline and TCP) in our previous study [30], it is important to re-examine them in the context of the current study. As shown in Figure 2, there was a tendency of increase in blood 5HT concentrations after TCP treatment (males 959 (1238, 718) ng/mL, females 927 (1478, 558) ng/mL) in comparison to saline (males 711 (1005, 242) ng/mL, females 349 (1069, 176) ng/mL) and 5HTP (males 484 (794, 188) ng/mL, females 827 (1277, 600) ng/mL) treatment. However, two-way ANOVA did not show significant effects of treatment (F(2,24) = 2.396; *p* = 0.1125), sex (F(1,24) = 0.7624; *p* = 0.3912), or treatment × sex interaction (F(2,24) = 1.156; *p* = 0.3318) on 5HT concentrations in the blood of adult animals.

### 3.2. Histomorphological and Gene Expression Changes in Jejunum

Histological sections of the jejunum viewed under a light microscope showed normal architecture in all layers of the digestive tract. All examined samples from the three experimental groups exhibited intact epithelial lining of the mucosa, well-preserved cellular integrity, and no evidence of distortion or crypt atrophy in the mucosa or signs of inflammation. Consequently, histomorphometric characteristics of digestive tract layers showed similar mean values for the measured parameters in all three groups (Table 1). Indeed, as analyzed by two-way ANOVA, treatment, sex, and treatment × sex interaction did not have a significant effect on either the epithelial layer width (F(2,30) = 2.08, *p* = 0.14; F(1,30) = 0.47, *p* = 0.4978 and F(2,30) = 0.33, *p* = 0.7181, respectively) or the muscle layer width (F(2,30) = 0.14, *p* = 0.8732; F(1,30) = 1.00, *p* = 0.3242 and F(2,30) = 0.39, *p* = 0.6775, respectively). The mucosal layer width was significantly affected by treatment × sex interaction (F(2,30) = 4.01, *p* = 0.0274), but no effect of treatment or sex (F(2,30) = 1.0, *p* = 0.3682 and F(1,30) = 0.029, *p* = 0.8653, respectively) was observed. 

**Figure 3:**
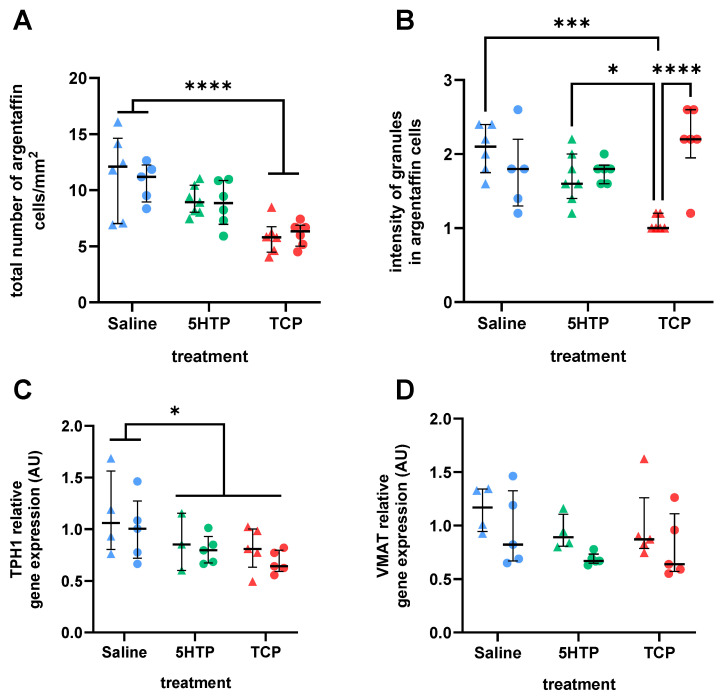
Effects of chronic treatment with serotonin enhancers 5-hydroxytryptophan (5HTP, 25 mg/kg) and tranylcypromine (TCP, 2 mg/kg) or saline on the small intestine (jejunum). (**A**) Number of argentaffin-positive cells, which produce serotonin, per mm^2^ of surface in the epithelial layer of the jejunum. Two-way ANOVA revealed a significant effect of treatment. (**B**) Staining intensity corresponding to the number of granules in argentaffin cells. Cells were rated subjectively from 1 (low intensity) to 3 (high intensity), and averages of 5 measurements per animal are shown. Two-way ANOVA revealed the significant effect of treatment × sex interaction. (**C**) Relative expression of tryptophan hydroxylase (TPH1) mRNA. Two-way ANOVA revealed the significant effect of treatment. (**D**) Relative expression of vesicular monoamine transporter (VMAT) mRNA. Results are shown as the median with interquartile range; males are depicted as triangles, and females as circles. * *p* < 0.05, *** *p* < 0.001, **** *p* < 0.0001, Tukey’s multiple comparison test for post hoc analyses.

An extremely significant effect of treatment was observed on the total number of argentaffin-positive cells (F(2,30) = 17.53, *p* < 0.0001), with no effects of sex (F(1,30) = 0.11, *p* = 0.7388) or treatment × sex interaction (F(21,30) = 0.15, *p* = 0.8621). As can be seen in Figure 3A, post hoc analysis revealed significantly lower values in the TCP-treated group in comparison to controls, with intermediate values of 5HTP-treated animals. Interestingly, the total number of argentaffin-positive cells significantly negatively correlated with blood 5HT concentration (r = −0.465, *p* = 0.01). The argentaffin-positive cells were stained brownish to black, with varying intensity depending on the number of granules within cells. The staining intensity of argentaffin cells was not significantly affected by treatment (F(2,30) = 1.98, *p* = 0.1558), although a significant effect was observed for both sex (F(1,30) = 5.864, *p* = 0.0217) and sex x treatment interaction (F(2,30) = 11.92, *p* = 0.0002). Post hoc analysis showed that this significance was due to males from the TCP treatment group having a significantly lower staining intensity than that of animals from the other subgroups (Figure 3B).

Treatment (F(2,21) = 4.013, *p* = 0.0334), but not sex (F(1,21) = 1.362, *p* = 0.2562) or treatment × sex interaction (F(2,21) = 0.05219, *p* = 0.9493), had a significant effect on the relative gene expression for TPH1. Post hoc analysis showed significantly lower mean levels of mRNA for TPH1 in both the treatment groups compared to the controls (Figure 3C). Although treatment (F(2,22) = 1.908, *p* = 0.1722, Figure 3D), as well as sex and treatment × sex interaction (F(1,22) = 4.192, *p* = 0.0527; and F(2,22) = 0.03419, *p* = 0.9664, respectively), did not significantly affect mRNA levels for *Vmat*, relative expression of this gene significantly positively correlated with the expression of the *Tph1* gene (r = 0.440, *p* = 0.022).

### 3.3. Histomorphological and Gene Expression Changes in Liver

**Figure 4:**
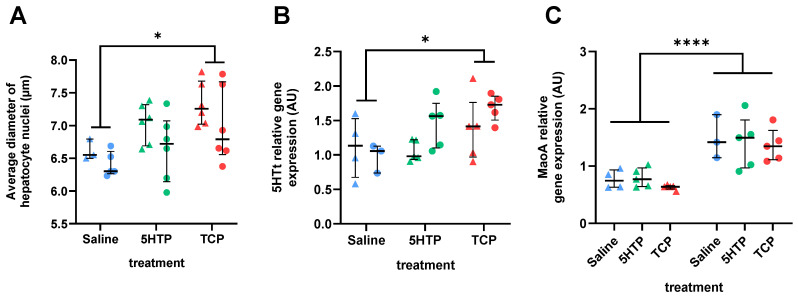
Effects of chronic treatment with serotonin enhancers 5-hydroxytryptophan (5HTP, 25 mg/kg) and tranylcypromine (TCP, 2 mg/kg) or saline on the liver. (**A**) Average diameter of hepatocyte nuclei. Each measure represents the average of the measured width of 100 hepatocyte nuclei. Two-way ANOVA revealed a significant effect of treatment. (**B**) Relative gene expression for 5HT transporter (5HTt) mRNA. Two-way ANOVA revealed a significant effect of treatment. (**C**) Relative gene expression for monoamine oxidase A (MAOA) mRNA. Two-way ANOVA revealed a significant effect of sex. Results are shown as the median with interquartile range; males are depicted as triangles and females as circles. * *p* < 0.05, **** *p* < 0.0001, Tukey’s multiple comparison test.

Microscopically, liver sections from all three groups appeared normal, with no heavy pathological findings such as inflammation, fat deposits, fibrosis, or necrosis. The hepatic parenchyma was organized into polygonal lobules with hepatocytes, normal portal tracts, and a regular pattern of the central veins. However, quantitative morphometric parameters revealed some changes in the treated groups. According to two-way ANOVA, treatment had a significant (F(2,26) = 6.259, *p* = 0.006) effect on hepatocyte nuclei diameter, with the values of TCP-treated group being significantly higher and the values of the 5HTP group being intermediate as compared to the values of the control group (Figure 4A). Sex had a marginally significant effect (F(1,26) = 4.399, *p* = 0.0458), and sex × treatment interaction had no effect (F(2,27) = 0.1107, *p* = 0.8956). Interestingly, the effect of treatment on nuclear enlargement (karyomegaly) was not followed by enlargement of hepatocytes (hepatocellular hypertrophy). Two-way ANOVA revealed only a significant effect of sex (F(1,27) = 9.252, *p* = 0.0052) but not of treatment or sex × treatment interaction on hepatocyte diameter (F(2,27) = 1.556, *p* = 0.2292 and F(2,27) = 1.031, *p* = 0.3703, respectively). 

Although some early signs of connective tissue deposition around the central veins (perivenular fibrosis) were visible in females treated with 5HTP, no significant differences between the treated groups were detected with two-way ANOVA for structural dilatation of the sinusoid diameter or central veins.

Treatment had a significant effect on the gene expression for 5HTt (F(2,21) = 4.636, *p* = 0.0215) due to a significantly higher expression in the TCP-treated group, with the values of the 5HTP-treated group again being in between (Figure 4B). No effect of sex (F(1,21) = 2.149, *p* = 0.1575) or sex × treatment interaction (F(2,21) = 1.453, *p* = 0.2565) was observed. A strong effect of sex was noticed for MAOA mRNA expression (F(1,21) = 38.69, *p* < 0.0001), with no effect of treatment or treatment × sex interaction (F(2,21) = 0.5400, *p* = 0.5906 and F(2,22) = 2.599, *p* = 0.0970; respectively).

We studied the long-lasting effects of perinatal exposure to excessive 5HT concentrations on the organs responsible for the maintenance of peripheral 5HT homeostasis. Animals treated perinatally with 5HT enhancers displayed decreased number and function of serotonin-producing cells in the jejunum, enlarged nuclei of the liver cells, and an increase in 5HTt mRNA expression. In comparison to TCP, 5HTP had smaller yet visible effects on the measured parameters.

## 4. Discussion

We showed earlier that blood serotonin levels were significantly increased in both treatment groups during treatment, while TCP, unlike 5HTP, also caused a significant increase in brain 5HT concentrations [26]. TCP seemed to have induced a more prominent disbalance in 5HT homeostasis, which affected both 5HT compartments, and we therefore expected to see more serious effects in TCP-treated animals than in 5HTP-treated animals. At adult age, differences in blood 5HT levels of the TCP- and 5HTP-treated animals were not significantly different from those in saline-treated rats, but a tendency of gradual saline-5HTP-TCP increase was still observed. The fact that 5HT levels did not fully return to normal seven weeks after the end of treatment indicates that peripheral 5HT homeostasis was not fully reestablished in spite of the compensatory mechanisms. Indeed, we observed specific, 5HT-related changes, which is in accordance with the reported findings that high levels of neurotransmitters during development induce permanent changes at the histological and cellular level [38,39].

As expected, perinatal treatment with 5HT enhancers did not have a gross effect on the enteric tissue, as the thickness of tunica mucosa, height of the mucosal epithelial cells, and thickness of the outer muscle wall remained unchanged. Argentaffin-positive cells from our study were pyramidal or triangular in shape (Figure 1A–C), similar to the description by Gustafsson et al. [40], and clearly showed the presence of secretory granules in the basal portion of the cells, which can activate vagal afferent fibers inducing intestinal motility and modulating brain–gut axis signaling, or they can be released into the cardiovascular system and stimulate glucose and lipid metabolism [41]. The TCP-treated group from our study showed a significantly lower number of argentaffin-positive cells and a lower presence of 5HT in the granules in these cells (significant only for males), which was reflected in the lower level of gene expression for TPH1 (significantly so in both the TCP- and 5HTP-treated groups). Although mRNA levels for VMAT1 significantly positively correlated with mRNA levels for TPH1, as it would be expected that less synthesis leads to less need for storage, the expression of this gene was not significantly lowered in the treated groups. Takahashi and colleagues [42] found that heterozygous *Vmat2* knock-out mice had reduced neuronal 5HT concentrations probably due to the lack of storage and, therefore, easier availability for degradation. A more stable concentration of VMAT than of TPH1 might be explained by an attempt to compensate for the decrease in the number of cells and synthesis rate and to protect the remaining 5HT from degradation. We assume that the reduction in number of 5HT-producing cells and down-regulation of *Tph1* expression, long after a washout-period, are the results of the extensive compensatory mechanism that occurred during the increased availability of the immediate 5HT precursor (in the case of 5HTP treatment) or the lack of degradation of 5HT during MAO inhibition (in the case of TCP treatment). In contrast with these results, a high-tryptophan diet during fetal and early postnatal development, caused, besides hyperserotonemia, an increase in the number of serotonin-producing cells [43]. The discrepancy between these results could be explained by the type of administered 5HT enhancer—5HT precursor tryptophan requires TPH1 as the rate-limiting enzyme crucial for the first step of serotonin synthesis, which might have caused an increase in both *Tph1* gene expression and the number of TPH1-expressing cells. In our experiment, there was no need for increased TPH1 activity, allowing for the compensatory reduction of serotonin-producing cells and enzymes.

As in the jejunum, changes at the tissue level were also absent in the liver—both sinusoid and central vein diameters showed no difference between the groups. At the cellular level, the hepatocyte nuclei diameter showed an increase in the treated groups paralleled by an increase in *5HTt* gene expression. The increase in both parameters was significant in the TCP-treated group with intermediate values in the 5HTP-treated group. A highly significant difference in *MaoA* expression between the sexes, with females having double the expression than males, was expected due to the location of this gene on the X chromosome. Interestingly, our earlier study demonstrated a marked, long-lasting compensatory increase in mRNA expression for MAOA and 5HTt in the brains of TCP-treated animals [28], yet no changes were observed in the liver *MaoA* gene expression after 5HT enhancement. Although we cannot rule out an increased *MaoA* expression in the lungs (another site of 5HT degradation), it seems that in the liver, serotonin enhancement specifically upregulates *5HTt* expression. Serotonin promotes liver regeneration [44] yet may cause hepatotoxicity due to the generation of reactive oxygen species (ROS) during degradation by MAOA [45]. This might be the reason why we did not see an increase in the peripheral expression of *MaoA* after chronic inhibition since the compensatory mechanism of increasing *MaoA* expression might have a deleterious effect on liver cells due to excess ROS. Instead, 5HT uptake into hepatocytes increased (as demonstrated by increased *5HTt* expression), after which the excess of 5HT might have been converted to melatonin, as hepatocytes were shown to express serotonin N-acetyltransferase and hydroxyindole-O-methyl transferase [46]. Alternatively, hepatocyte 5HT content might have remained increased, potentially inducing karyomegaly, as serotonin was reported to mediate the induction of DNA synthesis in primary cultures of rat hepatocytes [47] and stimulate liver regeneration after hepatectomy in humans [48].

Taken all together, our current results indicate that in the periphery, a reduction in 5HT synthesis (lower number of 5HT-producing cells and decreased *Tph1* expression) coupled with an enhanced removal of 5HT from blood plasma (increased *5HTt* expression) are the main means of compensation for excessive blood 5HT levels. The resulting alteration in 5HT production might have induced dysregulation of 5HT signaling and caused impairments in 5HT-regulated peripheral functions, such as bone maintenance and leukocyte development and/or the sustainment previously observed in adult TCP-treated rats [30]. Future investigation of the gene expression of proinflammatory molecules and the expression of serotonin receptors in both tissues should provide a deeper understanding of the physiological changes in the digestive tract as a consequence of perinatal 5HT enhancement.

In humans, inadequate 5HT production leads to various disorders such as inflammatory bowel diseases (IBDs), celiac disease, and neuroendocrine disorders [41]. Studies on 5HT signaling in IBD show that the number of EC cells and the presence of 5HT in secretory granules can be either increased or decreased both in animal and human models [49,50,51,52]. These differences may arise from different model organisms of induced diseases and from the severity and location of the disease [52]. Serotonin’s role in gut inflammation is well known [49,53,54,55,56,57]. In studies, inflammation in the intestine was present in *Tph1* or *5HTt* KO models fully lacking the ability of 5HT synthesis or removal, respectively. The administration of 5HTP to *Tph1*^–/–^ mice increased the number of 5HT-expressing cells and intestinal histologic damage [58], while gastrointestinal inflammation was induced with 2,4,6-trinitrobenzene sulfonic acid (TNBS) in a *5HTt* KO model [59]. The lack of inflammation in the intestines of the 5HTP- and TCP-treated rats 2 months after the end of treatment might be the result of the compensatory decrease in intestine *Tph1* expression and a parallel increase in liver *5HTt* expression. Still, both 5HTP- and TCP-treated rats had previously shown a slower rate of increase in body mass compared to the saline-treated rats [24,25], pointing to a possible inadequacy in nutrient absorption and storage and/or consumption and rendering them a potential model for studying gastrointestinal and metabolic disorders. 

The fact that the period of 5HTP and TCP treatment used in our study corresponds to the second and third trimester of human pregnancy [13] provides another point of potential clinical relevance to our model. Selective serotonin reuptake inhibitors (SSRIs) are commonly used 5HT enhancers during pregnancy [60], and the consequences of developmental exposure to SSRIs have been thoroughly studied in both animal models and human populations [61,62]. On the other hand, the use of MAO inhibitors is restricted to SSRI-nonresponsive patients; hence, reports on the prenatal exposure to MAO inhibitors are limited. Even less is known about the prenatal exposure to 5HTP, which is often offered as a natural alternative to antidepressant drugs, and the versatility of the potential therapeutic effects, easy availability, and convenience of unsupervised use increase the probability of prenatal exposure [20]. Effects analogous to those observed in our model, such as impeded growth and potential behavioral and/or metabolic alterations, might occur in exposed humans, suggesting that systematic studies on the prenatal impact of these 5HT enhancers in the human population are needed.

Our study has several advantages. First, by employing the inhibition of catabolism or the stimulation of synthesis, we were able to study the consequences of 5HT metabolic alterations, which complemented previously reported studies focused on 5HT synaptic action, i.e., treatment with SSRI’s [63] and 5HT receptor agonists [64,65]. Second, parallel use of the two compounds, one altering 5HT homeostasis only in the peripheral 5HT compartment and the other altering 5HT homeostasis both peripherally and centrally, enabled us to study the relationship between the two compartments and to examine the contribution of central 5HT disbalance to the alterations in the peripheral compartment. Third, the use of 5HTP instead of tryptophan (Trp) as a 5HT precursor allowed us to avoid the rate-limiting step in the synthesis of serotonin and to mimic the effect of increased serotonin synthesis through the chosen 5HTP dose. As opposed to Trp, which is an essential amino acid with many functions in the body (more than 90% of Trp enters the kynurenine metabolic pathway, about 5% is metabolized through the indole pathway by the gut microbiota, and only the remaining Trp is used in the serotonin synthesis [66,67]), 5HTP is only found in the serotonin synthesis pathway and is quantitatively converted to 5HT [20]. Fourth, experimenting on both male and female animals enabled us to check for the potential sex differences in the vulnerability to disbalance in 5HT homeostasis and to avoid a sex-biased interpretation of the results. Finally, the low chronic doses used in this study ae similar to those taken by humans, enhancing the translatability of the obtained results.

We also have to mention several limitations of our study, which could affect the reliability and interpretation of the results. The major limitation of the study lies in the relatively small number of animals, which could have caused some false-negative results due to insufficient sample power. The number of females included in the experiment was determined from our previous experience and according to the 3R principle [32], but rather small litter sizes and the loss of several samples resulted in a suboptimal sample number, rendering our findings only as preliminary and requiring further confirmation. We should note that TCP, as a nonselective MAO inhibitor, also affects catecholamine metabolism, allowing for the possibility that other monoamines influenced our results. Still, unlike 5HT, catecholamines can enter an alternative degradation pathway through catechol-O-methyltransferase, which is present in the brain and peripheral tissues and increases its activity when MAO is blocked [68]. Accordingly, we showed earlier that TCP treatment raised central dopamine and noradrenaline concentrations to a considerably lower extent than did that of 5HT, while it did not affect peripheral catecholamine levels [27]. Finally, our study did not include analysis at the protein level, which would have enabled us to establish a link between gene expression and structural changes and provide a clearer view of the mild yet significant effects of the 5HT enhancers.

## 5. Conclusions

We have shown that the perinatal exposure to the increased 5HT concentrations induces long-lasting cellular and molecular changes in the two organs responsible for serotonin metabolism, which may have a negative impact on 5HT availability and, consequently, on the 5HT-regulated peripheral functions. Our current and previous results demonstrate a link between developmental abnormalities of serotonin homeostasis and 5HT-related molecular, neurochemical, structural, and functional changes in adult life, suggesting our rat model to be suitable for exploring the neurobiological substrates of vulnerability to behavioral and metabolic disorders, as well as for modeling the adverse effects of the prenatal exposure to 5HT enhancers in the human population. Future studies on our model should help unravel the role of serotonin in mediating gut–brain interplay as well as microbiota–host interactions and, hopefully, set paths for the improvement of early diagnostics and individualized therapy.

## Figures and Tables

**Table 1:** 5-hydorxytryptophan and tranylcypromine induced changes in peripheral organs.

		Saline				5-Hydroxytryptophan				Tranilcypromine			
		Female		Male		Female		Male		Female		Male	
	5HT Conc.	Median (Q1,Q3)	N	Median (Q1,Q3)	N	Median (Q1,Q3)	N	Median (Q1,Q3)	N	Median (Q1,Q3)	N	Median (Q1,Q3)	N
	(ng 5-HT/mL PRP)	349 (1069,176)	5	711 (1005,242)	5	827 (1277,600)	5	484 (794,188)	5	927 (1478,558)	4	959 (1238,718)	6
	**Gene expression (arbitrary units)**												
**Jejunum**	TPH1	1.00 (1.27,0.72)	5	1.06 (1.56,0.80)	4	0.80 (0.93,0.67)	5	0.85 (1.15,0.60)	3	0.64 (0.80,0.59)	5	0.81 (1.00,0.63)	5
	VMAT	0.82 (1.33,0.67)		1.17 (1.34,0.95)		0.67 (0.73,0.65)		0.89 (1.11,0.81)	4	0.64 (1.11,0.57)		0.87 (1.26,0.79)	
**Liver**	5HTt	1.06 (1.13,0.74)	3	1.13 (1.53,0.67)	4	1.56 (1.75,1.10)	5	0.98 (1.22,0.93)	5	1.73 (1.85,1.50)	5	1.41 (1.76,0.97)	5
	MAOA	1.42 (1.90,1.15)		0.75 (0.93,0.63)		1.50 (1.81,0.97)		0.77 (0.97,0.64)		1.35 (1.62,1.11)		0.64 (0.66,0.60)	
	**Histology**												
**Jejunum**	Mucosal layer width (µm)	685.9 (741.5,644.3)	5	688.8 (706.8,566.4)	6	756.6 (766.5,725.4)	5	653.4 (776.0,589.6)	7	624.2 (697.8,525.3)	6	739.3 (779.4,637.9)	6
	Muscle layer width (µm)	67.5 (111.4,60.0)		82.6 (96.3,70.8)		71.0 (82.0,67.2)		90.9 (95.2,74,4)		71.9 (104.7,66.9)		92.8 (105.8,67.7)	
	Epithelial layer width (µm)	33.1 (36.0,31.6)		31.1 (35.0,28.4)		34.3 (36.0,32.7)		33.7 (38.4,31.6)		31.8 (33.7,29.5)		31.2 (34.7,28.6)	
	Total number of argentaffin-positive cells/mm^2^	11.2 (12.2,8.9)		12.1 (14.6,7.0)		8.8 (10.9,7.0)		8.9 (10.4,8.0)		6.3 (6.9,5.0)		5.8 (6.7,4.5)	
	Number of cells in villi/1 mm^2^	5.66 (8.37,4.84)		7.63 (9.17,4.86)		5.38 (6.41,4.76)		5.70 (7.03,4.99)		4.38 (5.07,3.39)		3.70 (4.21,2.95)	
	Number of cells in the crypts/1 mm^2^	3.40 (6.24,2.79)		4.47 (5.20,2.31)		3.47 (4.41,2.19)		3.05 (3.22,2.42)		1.91 (2.15,1.62)		2.10 (2.53,1.58)	
	Argentaffin cell intensity	1.80 (2.20,1.30)		2.10 (2.40,1.75)		1.80 (1.85,1.60)		1.60 (2.00,1.40)		2.20 (2.60,1.95)		1.00 (1.20,1.00)	
**Liver**	Central vein diameter (µm)	109.6 (121.6,99.9)	5	98.7 (115.3,84.4)	3	145.4 (164.5,112.5)	5	114.7 (154.0,97.0)	6	81.4 (116.2,62.5)	6	109.1 (147.9,59.7)	6
	Hepatocyte nuclei diameter (µm)	6.30 (6.60,6.27)		6.55 (6.79,6.50)		6.72 (7.07,6.14)		7.09 (7.32,6.69)		6.79 (7.67,6.56)		7.26 (7.68,7.02)	
	Hepatocyte diameter (µm)	20.0 (20.9,19.6)		21.2 (21.3,20.7)		20.0 (21.0,19.0)		21.5 (22.3,19.9)		18.9 (20.1,17.8)		21.1 (21.7,20.4)	
	Sinusoid diameter (µm)	4.44 (5.36,3.81)		4.30 (5.87,4.05)		4.15 (4.63,4.00)		5.03 (5.27,4.48)		5.31 (6.08,4.93)		4.93 (6.72,4.22)	

## Data Availability

Data is available at Mendeley Data: Gračan, Romana; Blazevic, Sofia; Brižić, Matea; Hranilovic, Dubravka (2024), “Adult rat jejunum and liver histological measurements and gene expression after perinatal exposure 5-hydroxytryptophan and tranylcypromine”, Mendeley Data, V1, doi: 10.17632/c44rsbnz5v.1

## Supplementary Materials

The following supporting information can be downloaded at: https://www.mdpi.com/article/10.3390/biomedicines12020357/s1, Figure S1: Gel electrophoresis after RNA isolation from liver tissue; Figure S2: Gel electrophoresis after RNA isolation from jejunum tissue; Table S1: Purity and concentration (c) of isolated RNA-liver; Table S2: Purity and concentration of isolated RNA–jejunum.

## References

[1] Berger M., Gray J.A., Roth B.L. (2009). The Expanded Biology of Serotonin. Annu. Rev. Med..

[2] Pawlak D., Oksztulska-Kolanek E., Znorko B., Domaniewski T., Rogalska J., Roszczenko A., Michalina Brzó Ska M., Pryczynicz A., Kemona A., Pawlak K. (2016). The Association between Elevated Levels of Peripheral Serotonin and Its Metabolite—5-Hydroxyindoleacetic Acid and Bone Strength and Metabolism in Growing Rats with Mild Experimental Chronic Kidney Disease. PLoS ONE.

[3] Deutch A.Y., Roth R.H., Byrne J.H., Roberts J.L. (2004). Pharmacology and Biochemistry of Synaptic Transmission: Classic Transmitters. From Molecules to Networks. An Introduction to Cellular and Molecular Neuroscience.

[4] Keszthelyi D., Troost F.J., Masclee A.A.M. (2009). Understanding the Role of Tryptophan and Serotonin Metabolism in Gastrointestinal Function. Neurogastroenterol. Motil..

[5] Weihe E., Schäfer M.K.H., Erickson J.D., Eiden L.E. (1994). Localization of Vesicular Monoamine Transporter Isoforms (VMAT1 and VMAT2) to Endocrine Cells and Neurons in Rat. J. Mol. Neurosci..

[6] Watanabe H., Rose M., Kanayama Y., Shirakawa H., Aso H. (2017). Energy Homeostasis by the Peripheral Serotonergic System. Serotonin—A Chemical Messenger Between All Types of Living Cells.

[7] Watts S.W., Morrison S.F., Davis R.P., Barman S.M. (2012). Serotonin and Blood Pressure Regulation. Pharmacol. Rev..

[8] Gershon M.D., Tack J.A.N. (2007). The Serotonin Signaling System: From Basic Understanding to Drug Development for Functional GI Disorders. Gastroenterology.

[9] Gershon M.D. (2013). 5-Hydroxytryptamine (Serotonin) in the Gastrointestinal Tract. Curr. Opin. Endocrinol. Diabetes. Obes..

[10] Choi W., Namkung J., Hwang I., Kim H.H.H.H., Lim A., Park H.J., Lee H.W., Han K.-H.H., Park S.S., Jeong J.-S.S. (2018). Serotonin Signals through a Gut-Liver Axis to Regulate Hepatic Steatosis. Nat. Commun..

[11] Ruddell R., Mann D., Ramm G. (2008). The Function of Serotonin within the Liver. J. Hepatol..

[12] Davies K.R., Richardson G., Akmentin W., Acuff V., Fenstermacher J.D., Couraud P.-O., Scherman D. (1996). The Microarchitecture of Cerebral Vessels. Biology and Physiology of the Blood-Brain Barrier: Transport, Cellular Interactions, and Brain Pathologies.

[13] Kepser L.-J.J., Homberg J.R. (2015). The Neurodevelopmental Effects of Serotonin: A Behavioural Perspective. Behav. Brain Res..

[14] Hanswijk S.I., Spoelder M., Shan L., Verheij M.M.M., Muilwijk O.G., Li W., Liu C., Kolk S.M., Homberg J.R. (2020). Gestational Factors throughout Fetal Neurodevelopment: The Serotonin Link. Int. J. Mol. Sci..

[15] Booij L., Richard T., Szyf M., Benkelfat C. (2015). Genetic and Early Environmental Influences on the Serotonin System: Consequences for Brain Development and Risk for Psychopathology. J. Psychiatry Neurosci..

[16] Cai Y., Li X., Zhou H., Zhou J. (2022). The Serotonergic System Dysfunction in Diabetes Mellitus. Front. Cell. Neurosci..

[17] Asarian L., Geary N., Ahima R., Kelly J., Elmquist J.J., Flier J., Ainslie D., Morris M., Wittert G., Turnbull H. (2013). Sex Differences in the Physiology of Eating. Am. J. Physiol. Regul. Integr. Comp. Physiol..

[18] Roth B.L. (1994). Multiple Serotonin Receptors: Clinical and Experimental Aspects. Ann. Clin. Psychiatry.

[19] Terry N., Margolis K.G. (2017). Serotonergic Mechanisms Regulating the GI Tract: Experimental Evidence and Therapeutic Relevance. Handb. Exp. Pharmacol..

[20] Birdsall T.C. (1998). 5-Hydroxytryptophan: A Clinically-Effective Serotonin Precursor. Altern. Med. Rev..

[21] De Ponti F., Ponti D. (2004). Pharmacology of Serotonin: What a Clinician Should Know. Gut.

[22] Roth B.L. (2007). Drugs and Valvular Heart Disease. N. Engl. J. Med..

[23] Hinz M., Stein A., Uncini T. (2012). 5-HTP Efficacy and Contraindications. Neuropsychiatr. Dis. Treat..

[24] Blazevic S., Dolenec P., Hranilovic D. (2011). Physiological Consequences of Perinatal Treatment of Rats with 5-Hydroxytryptophan. Period. Biol..

[25] Blazevic S.A., Jurcic Z., Hranilovic D. (2010). Perinatal Treatment of Rats with MAO Inhibitor Tranylcypromine. Transl. Neurosci..

[26] Hranilovic D., Blazevic S., Ivica N., Cicin-Sain L., Oreskovic D. (2011). The Effects of the Perinatal Treatment with 5-Hydroxytryptophan or Tranylcypromine on the Peripheral and Central Serotonin Homeostasis in Adult Rats. Neurochem. Int..

[27] Blazevic S.A., Merkler M., Persic D., Hranilovic D. (2017). Chronic Postnatal Monoamine Oxidase Inhibition Affects Affiliative Behavior in Rat Pups. Pharmacol. Biochem. Behav..

[28] Blazevic S., Hranilovic D. (2013). Expression of 5HT-Related Genes after Perinatal Treatment with 5HT Agonists. Transl. Neurosci..

[29] Blazevic S., Colic L., Culig L., Hranilovic D. (2012). Anxiety-like Behavior and Cognitive Flexibility in Adult Rats Perinatally Exposed to Increased Serotonin Concentrations. Behav. Brain Res..

[30] Blazevic S., Erjavec I., Brizic M., Vukicevic S., Hranilović D. (2015). Molecular Background and Physiological Consequences of Altered Peripheral Serotonin Homeostasis in Adult Rats Perinatally Treated with Tranylcypromine. J. Physiol. Pharmacol..

[31] Krinke G.J. (2000). The Laboratory Rat.

[32] Hubrecht R.C., Carter E. (2019). The 3Rs and Humane Experimental Technique: Implementing Change. Animals.

[33] Franklin T.B., Linder N., Russig H., Thöny B., Mansuy I.M. (2011). Influence of Early Stress on Social Abilities and Serotonergic Functions across Generations in Mice. PLoS ONE.

[34] Walker S.C., McGlone F.P. (2013). The Social Brain: Neurobiological Basis of Affiliative Behaviours and Psychological Well-Being. Neuropeptides.

[35] Goel N., Bale T.L. (2010). Sex Differences in the Serotonergic Influence on the Hypothalamic- Pituitary-Adrenal Stress Axis. Endocrinology.

[36] Pfaffl M.W. (2007). Relative Quantification. Real-Time PCR.

[37] Bustin S.A., Benes V., Garson J.A., Hellemans J., Huggett J., Kubista M., Mueller R., Nolan T., Pfaffl M.W., Shipley G.L. (2009). The MIQE Guidelines: Minimum Information for Publication of Quantitative Real-Time PCR Experiments. Clin. Chem..

[38] Di Pino G., Moessner R., Lesch K.-P., Lauder J.M., Persico A.M. (2004). Roles for Serotonin in Neurodevelopment: More than Just Neural Transmission. Curr. Neuropharmacol..

[39] Herlenius E., Lagercrantz H. (2001). Neurotransmitters and Neuromodulators during Early Human Development. Early Hum. Dev..

[40] Gustafsson B.I., Bakke I., Tømmerås K., Waldum H.L. (2006). A New Method for Visualization of Gut Mucosal Cells, Describing the Enterochromaffin Cell in the Rat Gastrointestinal Tract. Scand. J. Gastroenterol..

[41] Rezzani R., Franco C., Franceschetti L., Gianò M., Favero G. (2022). A Focus on Enterochromaffin Cells among the Enteroendocrine Cells: Localization, Morphology, and Role. Int. J. Mol. Sci..

[42] Takahashi N., Miner L.L., Sora I., Ujike H., Revay R.S., Kostic V., Jackson-Lewis V., Przedborski S., Uhl G.R. (1997). VMAT2 Knockout Mice: Heterozygotes Display Reduced Amphetamine-Conditioned Reward, Enhanced Amphetamine Locomotion, and Enhanced MPTP Toxicity. Proc. Natl. Acad. Sci. USA.

[43] Musumeci G., Loreto C., Trovato F.M., Giunta S., Imbesi R., Castrogiovanni P. (2014). Serotonin (5HT) Expression in Rat Pups Treated with High-Tryptophan Diet during Fetal and Early Postnatal Development. Acta Histochem..

[44] Lesurtel M., Graf R., Aleil B., Walther D.J., Tian Y., Jochum W., Gachet C., Bader M., Clavien P.-A.A. (2006). Platelet-Derived Serotonin Mediates Liver Regeneration. Science.

[45] Nocito A., Dahm F., Jochum W., Jang J.H., Georgiev P., Bader M., Renner E.L., Clavien P.A. (2007). Serotonin Mediates Oxidative Stress and Mitochondrial Toxicity in a Murine Model of Nonalcoholic Steatohepatitis. Gastroenterology.

[46] Myöhänen T.T., Schendzielorz N., Männistö P.T. (2010). Distribution of Catechol-O-Methyltransferase (COMT) Proteins and Enzymatic Activities in Wild-Type and Soluble COMT Deficient Mice. J. Neurochem..

[47] Balasubramanian S., Paulose C.S. (1998). Induction of DNA Synthesis in Primary Cultures of Rat Hepatocytes by Serotonin: Possible Involvement of Serotonin S2 Receptor. Hepatology.

[48] Padickakudy R., Pereyra D., Offensperger F., Jonas P., Oehlberger L., Schwarz C., Haegele S., Assinger A., Brostjan C., Gruenberger T. (2017). Bivalent Role of Intra-Platelet Serotonin in Liver Regeneration and Tumor Recurrence in Humans. J. Hepatol..

[49] Linden D.R., Chen J.X., Gershon M.D., Sharkey K.A., Mawe G.M. (2003). Serotonin Availability Is Increased in Mucosa of Guinea Pigs with TNBS-Induced Colitis. Am. J. Physiol. Gastrointest. Liver Physiol..

[50] Khan W.I., Motomura Y., Wang H., El-Sharkawy R.T., Verdu E.F., Verma-Gandhu M., Rollins B.J., Collins S.M. (2006). Critical Role of MCP-1 in the Pathogenesis of Experimental Colitis in the Context of Immune and Enterochromaffin Cells. Am. J. Physiol. Gastrointest. Liver Physiol..

[51] Xu X., Chen R., Zhan G., Wang D., Tan X., Xu H. (2021). Enterochromaffin Cells: Sentinels to Gut Microbiota in Hyperalgesia?. Front. Cell. Infect. Microbiol..

[52] Koopman N., Katsavelis D., Ten Hove A.S., Brul S., de Jonge W.J., Seppen J. (2021). The Multifaceted Role of Serotonin in Intestinal Homeostasis. Int. J. Mol. Sci..

[53] Banskota S., Khan W.I. (2022). Gut-Derived Serotonin and Its Emerging Roles in Immune Function, Inflammation, Metabolism and the Gut-Brain Axis. Curr. Opin. Endocrinol. Diabetes Obes..

[54] Wu H., Denna T.H., Storkersen J.N., Gerriets V.A. (2019). Beyond a Neurotransmitter: The Role of Serotonin in Inflammation and Immunity. Pharmacol. Res..

[55] Pergolizzi S., Alesci A., Centofanti A., Aragona M., Pallio S., Magaudda L., Cutroneo G., Lauriano E.R. (2022). Role of Serotonin in the Maintenance of Inflammatory State in Crohn’s Disease. Biomedicines.

[56] González Delgado S., Garza-Veloz I., Trejo-Vazquez F., Martinez-Fierro M.L. (2022). Interplay between Serotonin, Immune Response, and Intestinal Dysbiosis in Inflammatory Bowel Disease. Int. J. Mol. Sci..

[57] Ghia J.E., Li N., Wang H., Collins M., Deng Y., El-Sharkawy R.T., Côté F., Mallet J., Khan W.I. (2009). Serotonin Has a Key Role in Pathogenesis of Experimental Colitis. Gastroenterology.

[58] Bischoff S.C., Mailer R., Pabst O., Weier G., Sedlik W., Li Z., Chen J.J., Murphy D.L., Gershon M.D. (2009). Role of Serotonin in Intestinal Inflammation: Knockout of Serotonin Reuptake Transporter Exacerbates 2,4,6-Trinitrobenzene Sulfonic Acid Colitis in Mice. Am. J. Physiol. Gastrointest. Liver Physiol..

[59] Jørandli J.W., Thorsvik S., Skovdahl H.K., Kornfeld B., Sæterstad S., Gustafsson B.I., Sandvik A.K., Van Beelen Granlund A. (2020). The Serotonin Reuptake Transporter Is Reduced in the Epithelium of Active Crohn’s Disease and Ulcerative Colitis. Am. J. Physiol. Gastrointest. Liver Physiol..

[60] Alwan S., Friedman J.M. (2009). Safety of Selective Serotonin Reuptake Inhibitors in Pregnancy. CNS Drugs.

[61] Homberg J.R., Schubert D., Gaspar P. (2010). New Perspectives on the Neurodevelopmental Effects of SSRIs. Trends Pharmacol. Sci..

[62] Udechuku A., Nguyen T., Hill R., Szego K. (2010). Antidepressants in Pregnancy: A Systematic Review. Aust. N. Z. J. Psychiatry.

[63] Glover M.E., Clinton S.M. (2016). Of Rodents and Humans: A Comparative Review of the Neurobehavioral Effects of Early Life SSRI Exposure in Preclinical and Clinical Research. Int. J. Dev. Neurosci..

[64] Whitaker-Azmitia P.M. (2005). Behavioral and Cellular Consequences of Increasing Serotonergic Activity during Brain Development: A Role in Autism?. Int. J. Dev. Neurosci..

[65] Cannizzaro C., Plescia F., Gagliano M., Cannizzaro G., Provenzano G., Mantia G., Cannizzaro E. (2007). Effects of Pre- and Postnatal Exposure to 5-Methoxytryptamine and Early Handling on an Object-Place Association Learning Task in Adolescent Rat Offspring. Neurosci. Res..

[66] Hou Y., Li J., Ying S. (2023). Tryptophan Metabolism and Gut Microbiota: A Novel Regulatory Axis Integrating the Microbiome, Immunity, and Cancer. Metabolites.

[67] Tanaka M., Szabó Á., Spekker E., Polyák H., Tóth F., Vécsei L. (2022). Mitochondrial Impairment: A Common Motif in Neuropsychiatric Presentation? The Link to the Tryptophan–Kynurenine Metabolic System. Cells.

[68] Mannisto P.T., Kaakkola S. (1999). Catechol-O-Methyltransferase (COMT): Biochemistry, Molecular Biology, Pharmacology, and Clinical Efficacy of the New Selective COMT Inhibitors. Pharmacol. Rev..

