# Supplementary material for: Beyond the Brain: Perinatal Exposure of Rats to Serotonin Enhancers Induces Long-Term Changes in the Jejunum and Liver
Source: Biomedicines. 2024 Feb 3;12(2):357. doi: 10.3390/biomedicines12020357 (PMC10887406; doi:10.3390/biomedicines12020357)
Supplement: Supplementary file 1 [file biomedicines-12-00357-s001.zip › biomedicines-2812470-supplementary.pdf]

Supplementary material to Gračan et al.: ***Beyond the brain: perinatal exposure of rats to serotonin enhancers induces long-term changes in jejunum and liver***

Tissue samples were collected for histological and gene expression analysis from the second part of the small intestine (jejunum) and from the left liver lobe. For mRNA expression analysis, approximately 85 mg of the liver and 65 mg of jejunum tissue was immediately cut with a scalpel, washed in cold saline, placed in microtubes and frozen in liquid nitrogen. Total cellular RNA was isolated from liver and jejunum samples of 37 rats. Samples were disrupted and homogenized with an ultrasonic homogenizer (Bandelin electronic, Mecklenburg-Vorpommern, Germany) in 500 µL of guanidinium thiocyanate solution and frozen at –80°C until further processing. RNA isolation was performed utilizing the phenol-free RNAqueous-4PCR kit (Ambion, Inc., Austin, TX, USA), following the manufacturer's guidelines. Subsequently, genomic DNA was eliminated as per the provided instructions.

RNA quality and concentrations were assessed through agarose gel (1,5%) electrophoresis. The presence of bands with a size of about 5 kb, which corresponds to 28S rRNA, and about 2 kb, which corresponds to 18S rRNA, in an approximate ratio of 2:1, indicates satisfactory integrity of RNA molecules. Representative photographs of agarose gel visualization using a UV transilluminator after electrophoretic separation of RNA isolated from liver tissue (Figure S1) and jejunum (Figure S2) are shown. The presence of both bands in the wells shows the integrity of isolated RNA. We determined the purity and concentration of the isolated RNA using a spectrophotometer (Libra S51; Biochrom, UK). Tables show the results of the measured absorbance at wavelengths 260 and 280 nm, purity, and concentration (c) of the RNA samples from liver (Table S1) and jejunum (Table S2) corresponding to the gel electrophoresis in Figures S1 and S2.

In Figure S1, well 2 shows no RNA was present in accordance with the lack of absorbance measurements seen in Table S1. In Figure S2, wells 1, 3, 4, 13 show degraded RNA which is not coupled with a lack of absorbance as seen in table S2. These results highlight the need for using gel electrophoresis to test for RNA integrity before continuing with downstream applications. These samples were excluded from further processing.

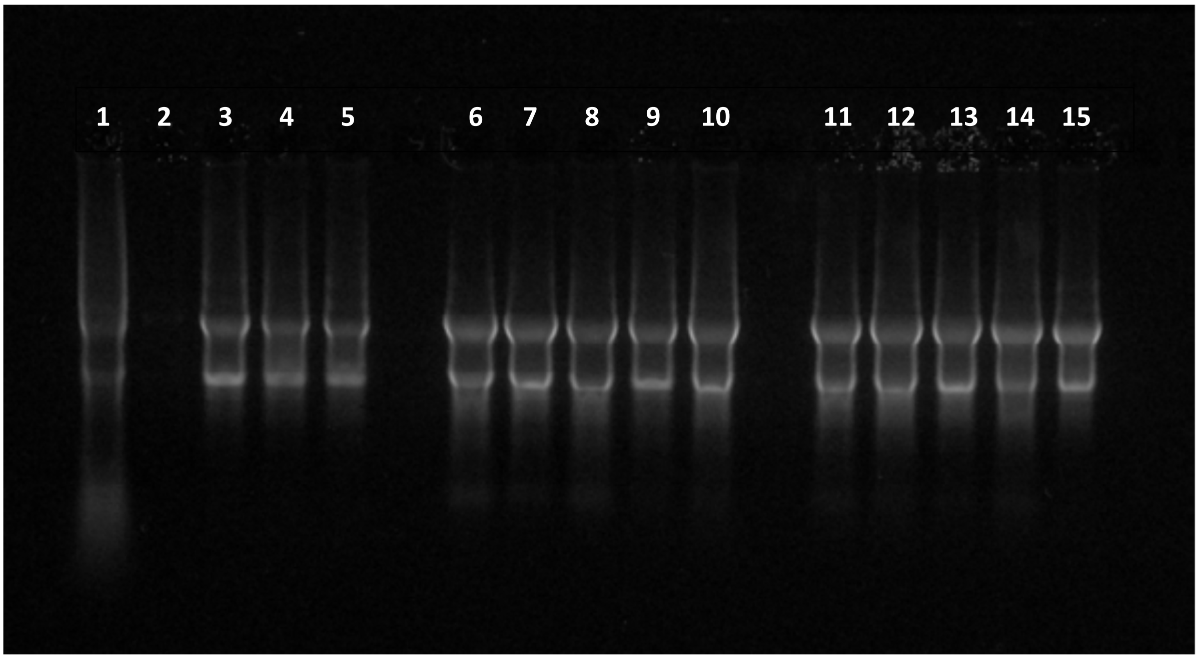

Figure S1. Gel electrophoresis after RNA isolation from liver tissue

Table S1. Purity and concentration (c) of isolated RNA - liver

	260	280	260/280	c (ng/µL)	observations
1	1,77	0,867	2,04	3910,82	
2	0,005	-0,002	-2,50	11,05	no RNA was present as confirmed in Figure S1
3	0,809	0,38	2,13	1787,49	
4	0,48	0,221	2,17	1060,56	
5	0,677	0,322	2,10	1495,83	
6	1,111	0,529	2,10	2454,75	
7	1,332	0,638	2,09	2943,05	
8	1,452	0,692	2,10	3208,19	
9	1,092	0,517	2,11	2412,77	
10	1,58	0,755	2,09	3491,01	
11	0,966	0,459	2,10	2134,38	
12	1,293	0,618	2,09	2856,88	
13	0,903	0,433	2,09	1995,18	
14	1,194	0,569	2,10	2638,14	
15	0,691	0,328	2,11	1526,76	

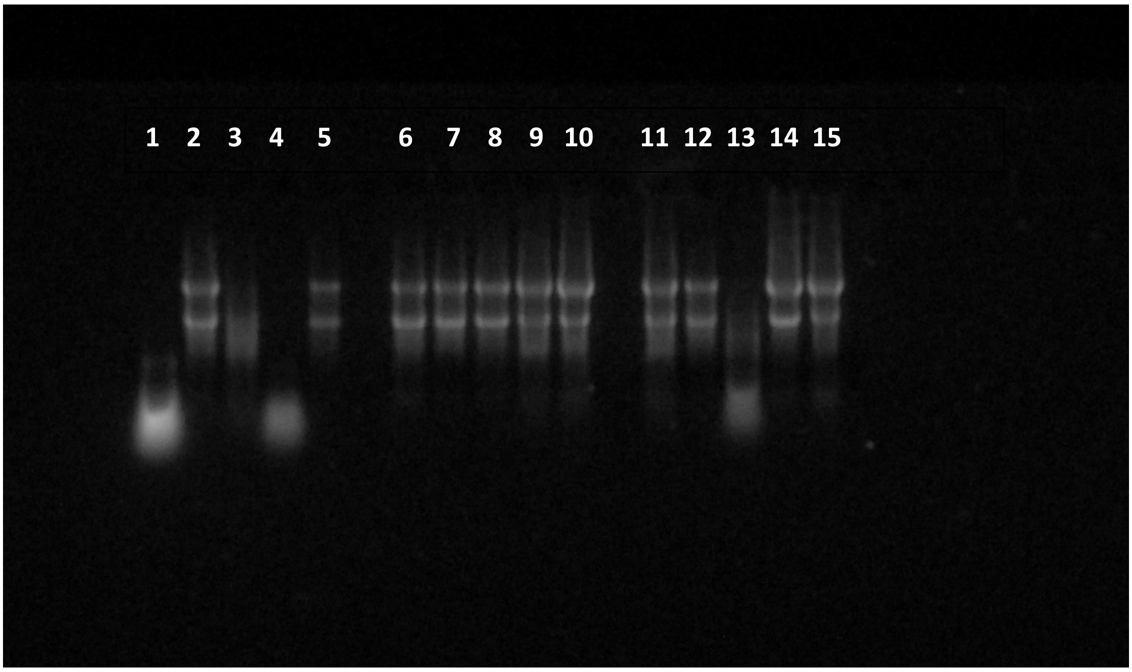

Figure S2. Gel electrophoresis after RNA isolation from jejunum tissue

Table S2. Purity and concentration of isolated RNA – jejunum

	260	280	260/280	c (ng/µL)	Observations
1	1,373	0,65	2,11	3033,64	Visible degradation in Figure S2
2	0,743	0,36	2,06	1641,66	
3	0,316	0,157	2,01	698,20	Visible degradation in Figure S2
4	0,411	0,198	2,08	908,10	Visible degradation in Figure S2
5	0,203	0,101	2,01	448,53	
6	0,649	0,316	2,05	1433,97	
7	0,463	0,232	2,00	1023,00	
8	0,65	0,304	2,14	1436,18	
9	0,991	0,479	2,07	2189,61	
10	1,115	0,538	2,07	2463,59	
11	0,883	0,426	2,07	1950,99	
12	0,345	0,167	2,07	762,28	
13	1,137	0,553	2,06	2512,20	Visible degradation in Figure S2
14	1,455	0,703	2,07	3214,82	
15	1,309	0,629	2,08	2892,24	
